# Oridonin protects against cardiac hypertrophy by promoting P21-related autophagy

**DOI:** 10.1038/s41419-019-1617-y

**Published:** 2019-05-24

**Authors:** Man Xu, Chun-xia Wan, Si-hui Huang, Hui-bo Wang, Di Fan, Hai-Ming Wu, Qing-qing Wu, Zhen-guo Ma, Wei Deng, Qi-Zhu Tang

**Affiliations:** 1 Department of Cardiology, Renmin Hospital of Wuhan University, Wuhan, 430060 China; 2 Cardiovascular Research Institute of Wuhan University, Wuhan, 430060 China; 3 Hubei Key Laboratory of Cardiology, Wuhan, 430060 China

**Keywords:** Macroautophagy, Target identification, Target validation, Cardiac hypertrophy

## Abstract

Autophagy is an endogenous protective process; the loss of autophagy could destabilize proteostasis and elevate intracellular oxidative stress, which is critically involved in the development of cardiac hypertrophy and heart failure. Oridonin, a natural tetracycline diterpenoid from the Chinese herb Rabdosia, has autophagy activation properties. In this study, we tested whether oridonin protects against cardiac hypertrophy in mice and cardiomyocytes. We implemented aortic banding to induce a cardiac hypertrophy mouse model, and oridonin was given by gavage for 4 weeks. Neonatal rat cardiomyocytes were stimulated with angiotensin II to simulate neurohumoural stress. Both in vivo and in vitro studies suggested that oridonin treatment mitigated pressure overload-induced cardiac hypertrophy and fibrosis, and also preserved heart function. Mice that received oridonin exhibited increased antioxidase activities and suppressed oxidative injury compared with the aortic banding group. Moreover, oridonin enhanced myocardial autophagy in pressure-overloaded hearts and angiotensin II-stimulated cardiomyocytes. Mechanistically, we discovered that oridonin administration regulated myocardial P21, and cytoplasmic P21 activated autophagy via regulating Akt and AMPK phosphorylation. These findings were further corroborated in a P21 knockout mouse model. Collectively, pressure overload-induced autophagy dysfunction causes intracellular protein accumulation, resulting in ROS injury while aggravating cardiac hypertrophy. Thus, our data show that oridonin promoted P21-related autophagic lysosomal degradation, hence attenuating oxidative injury and cardiac hypertrophy.

## Introduction

Cardiovascular diseases remain the leading cause of mortality worldwide^1^. Heart failure (HF) is the final common pathway of chronic cardiovascular disorders, and few therapies, including neurohormonal antagonists and approaches for symptomatic relief or inotropic action, have provided early interventions or positive long-term outcomes to date^2^. Cardiac hypertrophy has received considerable attention as a crucial pathophysiologic process of HF^3,4^. Haemodynamic overload, such as pressure overload, impels the heart to initiate various adaptive mechanisms, including hypertrophy to cope with wall stress increase, and altered protein synthesis to preserve myocyte survival and pump function. Long-lasting stress stimuli may elicit maladaptive alterations, such as disarranged autophagy, impaired protein homeostasis, oxidative stress injury and myocardial fibrosis^5,6^. Pharmacological therapies that modulate hypertrophic signalling may intervene in HF at the preliminary stage to avert hard-to-treat conditions and embody great promise for improving the prognosis of HF^7^.

As a vital mechanism for eliminating cytosolic misfolding proteins and delivering them to lysosomes for degradation and recycling, myocardial autophagy helps maintain the quality of the intracellular environment and conserves the ATP level in response to energy stress^8,9^. Previous studies showed that autophagy augmentation alleviates intracellular oxidative injury^10^. Increasing evidence, using genetic or pharmacological interventions, indicates that maintaining autophagy plays a protective role in the heart during cardiac remodelling, which limits myocardial damage in response to pressure overload^11–13^ or ischaemia^14^. Numerous pharmacological agents that can regulate autophagy have been identified. However, clinical applications of maintaining moderate autophagy are challenging due to the proper extent of autophagy activation and the side effects inherent in each intervention^15^. In view of this, new drugs that are most appropriate for upholding autophagy have yet to be identified in the treatment of cardiac diseases.

Traditional Chinese medicines have been widely and effectively used in treating cardiovascular diseases, and compounds from various herbs play important roles in taming diverse pathophysiological processes such as peroxidation and metabolic abnormalities^16^. Oridonin, a natural tetracycline diterpenoid, is a flavonoid compound extracted from the Chinese herb *Rabdosia rubescens*, which is known for its well-studied, multitargeting properties for antitumour activities^17^. Studies have also verified the anti-proliferation effects of oridonin^18^. Furthermore, oridonin exerts stimulative actions on autophagy^19,20^. Given the growth-inhibition and autophagy-promotion effects together, we propose the hypothesis that oridonin may play a role in cardiac hypertrophy.

The present study investigated the effects of oridonin on pressure overload induced cardiac hypertrophy and explored the underlying mechanisms. Our results confirmed that oridonin mitigated cardiac hypertrophy and preserved heart function. Mechanistically, we further discovered that oridonin-prevented maladaptive hypertrophy was partially dependent on P21 signalling-regulated myocardial autophagy.

## Results

### Oridonin inhibited cardiomyocyte hypertrophy

**Fig. 1:**
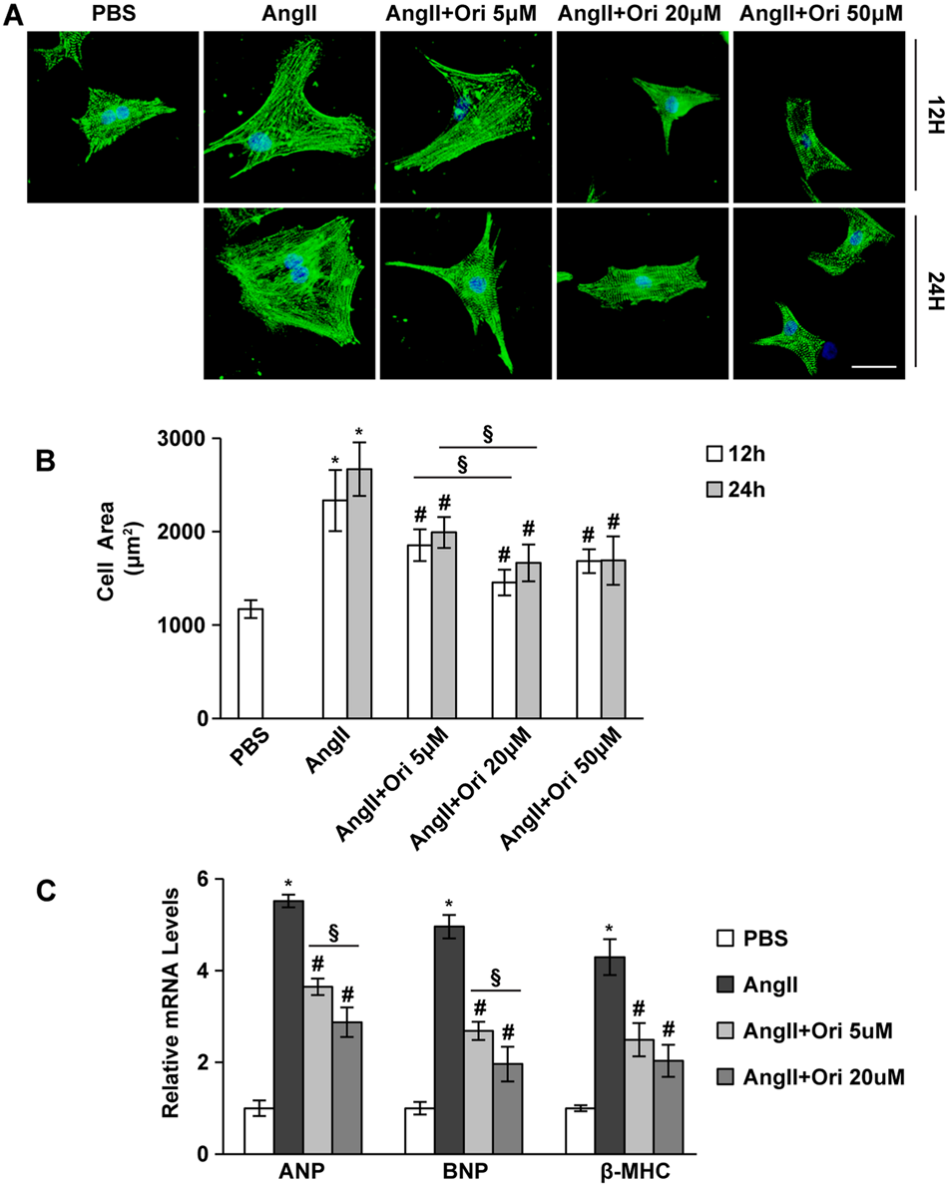
Oridonin inhibited cardiomyocyte hypertrophy. **a** A-actinin staining of the cardiomyocytes incubated with different doses of oridonin (5, 20 and 50 μM) in response to Ang II for 12–24 h. Representative images show the inhibitory effect of oridonin on the enlargement of myocytes (scale bar: 20 μm). **b** Statistical results of cell area by measuring random cells (*n* = 50 cells). **c** Real-time PCR analyses of the hypertrophy markers atrial natriuretic peptide (ANP), brain natriuretic peptide (BNP), and β-myosin heavy chain (β-MHC) (*n* = 6). Data are represented as the means ± SD. *P < 0.05 versus vehicle cells; #*P* < 0.05 versus Ang II-treated cells, §*P* < 0.05 between different doses of oridonin

To examine whether oridonin protects cardiomyocytes against stress-induced hypertrophy, we used primary cultured neonatal rat cardiomyocytes (NRCMs) in a well-controlled experimental setting. Cells were treated with Ang II for 12 or 24 h, and oridonin was used at the indicated concentrations that were previously verified to be safe (Fig. S1, *P* < 0.05). Cell size was assessed with α-actinin immunostaining. The results suggested that oridonin treatment patently diminished the increase in cardiomyocyte size in the presence of Ang II after 12 or 24 h of culture, and the inhibitory effects were seen in a dose-dependent manner ranging between 5 and 20 μM (Figs. 1a, b, *P* < 0.05). No discernible differences between 12 and 24 h at each dose were observed. In addition, oridonin markedly attenuated the increased transcription levels of atrial natriuretic peptide (ANP), B-type natriuretic peptide (BNP), and β-myosin heavy chain (β-MHC) provoked by Ang II, especially in the oridonin-20 μM group (Fig. 1c, *P* < 0.05). These data implied that oridonin could inhibit Ang II induced isolated cardiaomyocyte hypertrophy.

### Oridonin inhibited cardiac hypertrophy in pressure overload hearts

**Fig. 2:**
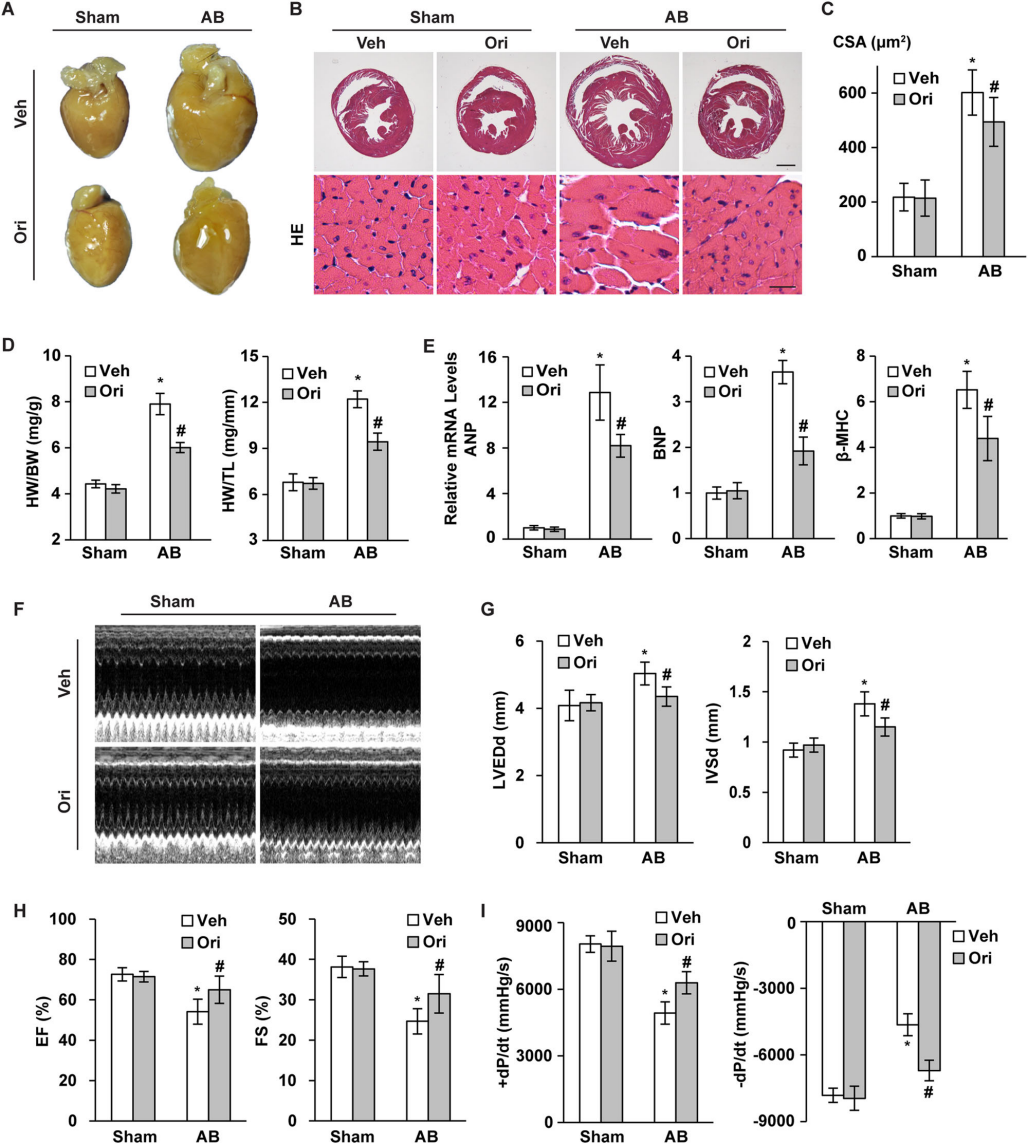
Oridonin ameliorated cardiac hypertrophy and dysfunction induced by pressure overload. **a**, **b** Histological analysis of whole hearts and heart sections stained with HE 4 weeks post AB surgery (scale bar: 1000 μm and 20 μm, *n* = 6). **c** Statistical results for the cross-sectional areas of myocytes (CSA, *n* = 100 cells). **d** Statistical results of heart weight/body weight (HW/BW) and heart weight/tibia length (HW/TL) (*n* = 12–15). **e** Real-time PCR analyses of the transcriptional levels of atrial natriuretic peptide (ANP), brain natriuretic peptide (BNP), and β-myosin heavy chain (β-MHC) (*n* = 6). **f** Representative M-mode echocardiograms of indicated groups 4 weeks after surgery (*n* = 6). **g**, **h** Echocardiographic parameters of each group of mice (*n* = 6). **i** Haemodynamic parameters in oridonin-treated mice (*n* = 6). Data are represented as the means ± SD. *P < 0.05 versus vehicle-sham. #*P* < 0.05 versus vehicle-AB

To demonstrate the beneficial role of oridonin in cardiac hypertrophy, we next sought to discover whether oridonin could attenuate the hypertrophic response induced by pressure overload. Mice were subjected to pressure overload by aortic banding (AB) surgery, and 40 mg/kg of oridonin was given orally once a day (Fig. S1). After 4 weeks of administration, mice under basal conditions did not show any alterations in cardiac structure or function with or without oridonin treatment. AB surgery resulted in greater ventricular wall thickness and inferior cardiac function. However, compared with the untreated AB mice, mice with the protection of oridonin treatment exhibited mitigatory cardiac hypertrophy, as shown by the gross appearance of the heart, the myocyte cross sectional area, the mass of the heart (HW/BW and HW/TL ratios), and the expression of hypertrophic markers (ANP, BNP, βMHC) (Figs. 2a–e, *P* < 0.05). Moreover, after AB surgery, left ventricular dilation occurs, oridonin could prevent the LV enlargement, presented by limited increase of LV end-diastolic dimension. The increase of interventricular septal thickness at end-diastole and decrease of ejection fraction and fractional shortening (FS), were also prevented by oridonin (Figs. 2f–h, *P* < 0.05). In parallel with this, harm in active relaxation was also prevented in oridonin-treated mice, as these mice displayed an increased rate of pressure development or decay (±dp/dt) compared with the AB group (Fig. 2i, *P* < 0.05).

### Oridonin attenuated pressure overload-induced cardiac fibrosis

Cardiomyocyte injury during hypertrophy promotes alterations within the extracellular matrix and induces fibrosis. To further define the effect of oridonin on cardiac fibrosis, picrosirius red (PSR) staining and quantitative analysis were performed. The results indicated that hearts subjected to chronic pressure overload developed significantly increased fibrosis in the interstitial and perivascular spaces compared with the sham operated hearts, and this fibrosis was remarkably limited in oridonin-treated hearts (Fig. S2a, b, *P* < 0.05). Consistently, 4 weeks after AB surgery, the expression levels of the markers related to cardiac fibrosis, including collagen Iα, collagen IIIα and CTGF, were lower in the hearts of oridonin treated mice compared with vehicle hearts (Fig. S2c, *P* < 0.05).

### Oridonin restrained cardiac oxidative stress

**Fig. 3:**
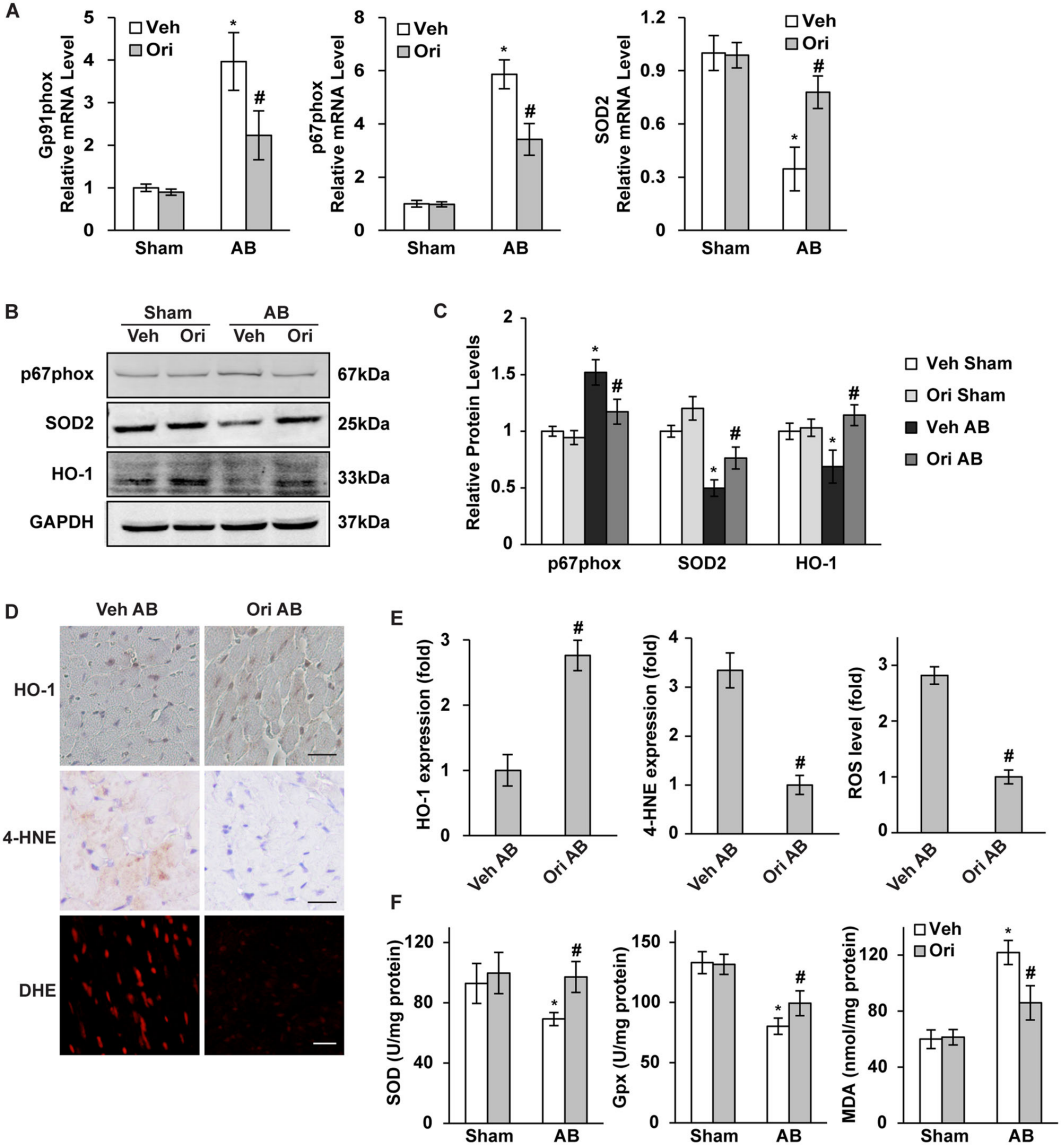
Oridonin inhibited pressure overload-induced oxidative stress injury. **a** Real-time PCR analyses of Gp91phox, p67phox and SOD2 (*n* = 6). **b**, **c** Immunoblotting and quantification analyses of p67phox, SOD2 and HO-1 (n = 6). **d**, **e** Immunohistochemical analyses of HO-1 and 4-HNE in hypertrophic hearts, and DHE detection of ROS level. (scale bar: 20 μm). **f** Total activities of antioxidants SOD and GPx, and lipid peroxidation MDA in mouse hearts (*n* = 6). *P < 0.05 versus vehicle-sham. #*P* < 0.05 versus vehicle-AB

Cardiac hypertrophy is always accompanied by excessive oxidative stress injury; hence, we evaluated the anti-oxidant effects of oridonin, the extent of which was detected by the expression of oxidase and lipid peroxidation, and the antioxidase activities. We first assessed the transcription levels of the NADPH oxidase subunits gp91phox, p67phox and SOD2. Compared with the AB group, the cardiac mRNA and serum levels of gp91phox, p67phox and SOD2 were all significantly decreased in the presence of oridonin (Fig. 3a, *P* < 0.05). Protein expression detected by WB showed similar alterations of p67phox and SOD2 as mRNA levels, and restored haem oxygenase 1 by oridonin treatment (Figs. 3b, c, *P* < 0.05). IHC staining showed the oridonin could raise the expression of myocardial HO-1 while limiting the level of 4-hydroxynonenal after AB surgery (Figs. 3d, e, *P* < 0.05). Dihydroethidium fluorescence showed oridonin could restrict the generation of ROS after AB (Figs. 3d, e, *P* < 0.05). Quantitative analysis showed enhanced myocardial SOD and GPx with oridonin treatment post AB, while the MDA level was reduced in oridonin-AB group (Fig. 3f, *P* < 0.05).

### Oridonin boosted myocardial autophagy in pressure-overloaded hearts and Ang II-stimulated cardiomyocytes

**Fig. 4:**
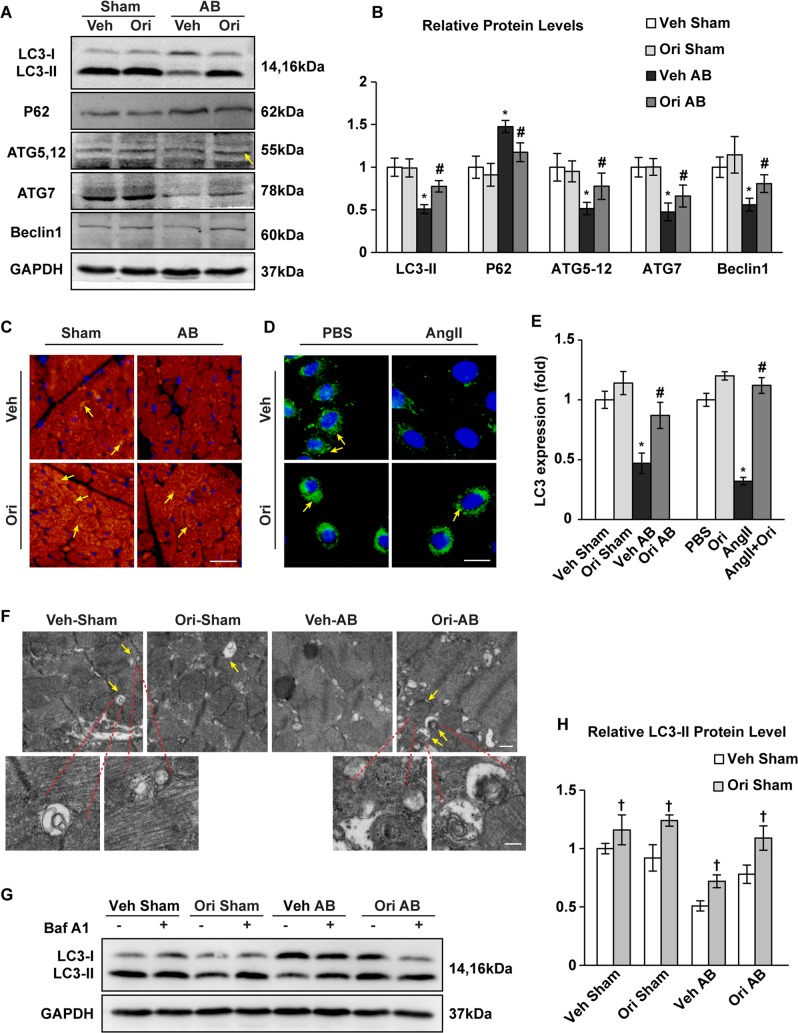
The effects of oridonin on myocardial autophagy. **a**, **b** Immunoblotting and quantification analyses of the protein levels of LC3-I/II, P62, Atg5-12, Atg7, and Beclin1 in oridonin- and vehicle-treated mice after 1 week of AB surgery (*n* = 6). **c**–**e** Detection of LC3 expression by immunofluorescence staining in the indicated mice after 1 week of AB (scale bar: 20 μm, *n* = 6), and in H9C2 cardiomyocytes (scale bar: 20 μm, *n* = 6). Arrows indicate LC3-II dots. *P < 0.05 versus vehicle-sham. #*P* < 0.05 versus vehicle-AB (left panel). *P < 0.05 versus vehicle-PBS. #P < 0.05 versus vehicle-AngII (right panel). **f** Representative transmission electron microscope images of cardiomyocytes 1 week post AB surgery. Arrows indicate autophagosomes and autolysosomes containing electron-dense contents. Scale bars: 500 nm (up) and 200 nm (down). **g**, **h** Relative LC3-II/GAPDH levels after Baf A1 intraperitoneal injection in mice (*n* = 6, ^†^*P* < 0.05)

Autophagy plays an essential role in maintaining cellular homeostasis in the heart, both under baseline and stress conditions. Exacerbated cardiac oxidative stress could also be the consequence of impaired autophagy. Autophagic activities after AB surgery were analysed by immunoblotting of a panel of autophagy-related genes, staining of protein-LC3 dots, and observing of autophagosomes and autolysosomes. Autophagy was suppressed in AB-induced hypertrophied hearts compared to sham operated hearts at 1-week post-surgery. In AB hearts treated with oridonin, LC3II expression was significantly increased, with increased ATG5-12, ATG7 and Beclin1 but decreased accumulation of P62 (Figs. 4a, b, *P* < 0.05). A similar conclusion was obtained by detection of LC3II accumulation using immunofluorescence staining both in mouse hearts and cardiomyocytes (Figs. 4c–e, *P* < 0.05). Transmission electron microscopy revealed infrequency of autophagosomes and autolysosomes in pressure overloaded hearts, whereas oridonin treatment increased the amount of autophagosomes and autolysosomes (Fig. 4f). Further more, we assessed autophagic flux in mice by intraperitoneal injection of BafA1. LC3-II levels were greatly improved after using BafA1, which showed the blockage of autophagic flux. Oridonin treatment did not alter the autophagic blockage effect of BafA1 (Fig. 4g, h, *P* < 0.05).

### Protective effects of oridonin involve Akt/AMPK and P21 signalling

**Fig. 5:**
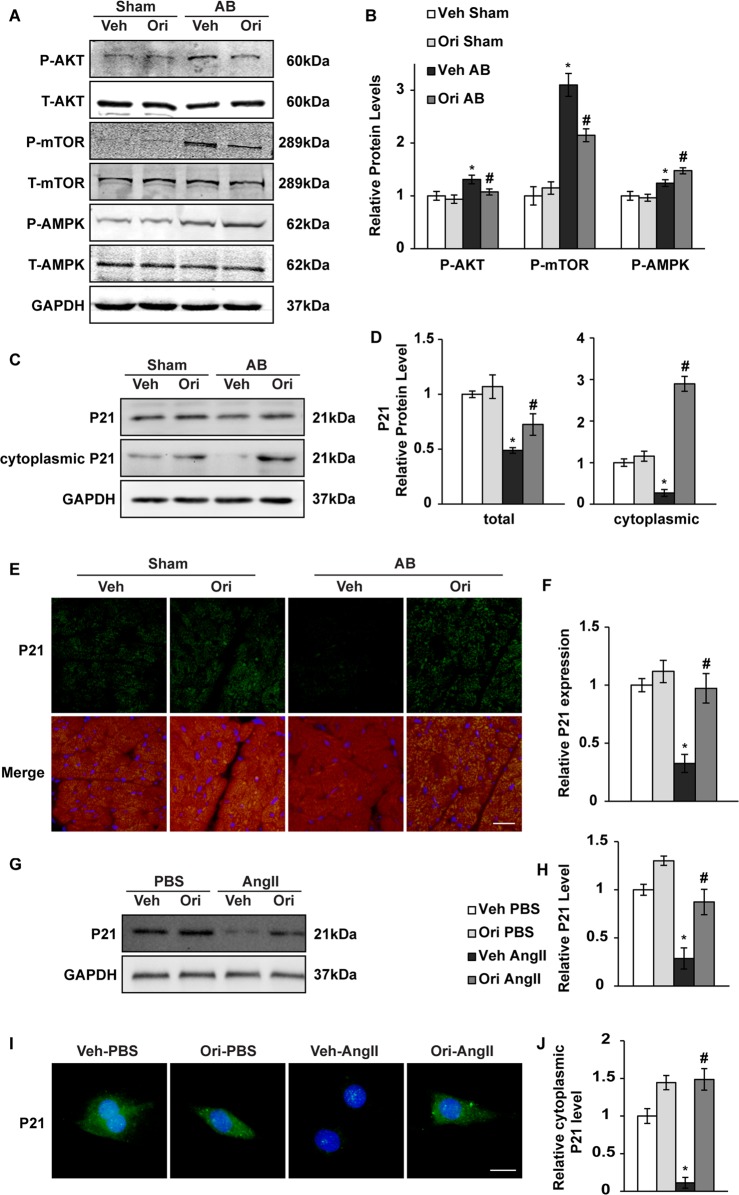
The effects of oridonin on AKT-mTOR-AMPKα and P21 signalling pathways. **a**, **b** Representative Western blot analyses of P-AKT, T-AKT, P-mTOR, T-mTOR, P-AMPK and T-AMPK (*n* = 6). **c**, **d** The relative protein expression level of P21 in heart tissue and cytoplasm (*n* = 6). **e**, **f** Immunofluorescence analysis of P21 in mouse hearts (scale bar: 20 μm, *n* = 6). *P < 0.05 versus vehicle-sham. #*P* < 0.05 versus vehicle-AB. **g**, **h** The relative protein expression level of P21 in NRCMs with Ang II stimulation (*n* = 6). **i**, **j** Immunofluorescence analysis of P21 in NRCMs (scale bar: 20 μm, *n* = 6). *P < 0.05 versus vehicle-PBS. #*P* < 0.05 versus vehicle-Ang II

To further study the role of oridonin in upholding autophagy, we primarily examined the classical signal pathways and molecules involved. MTOR complex 1 (mTORC1) and AMP-activated protein kinase (AMPK) are well established as upstream kinases that regulate autophagy during cardiac hypertrophy^21^. Phosphorylation (P-) of AKT and mTOR was remarkably increased after 1 week of pressure overload, which was blunted by oridonin treatment. Phosphorylation of AMPK, which is critical to autophagic action, was promoted by oridonin (Figs. 5a, b, *P* < 0.05). It has been demonstrated that upregulation of P21 is relevant to autophagy, as well as a target of oridonin^22^. Therefore, we inspected the protein level and distribution of P21. We found that P21 expression was down regulated by 1 week after AB, but promoted by oridonin, especially the alteration of cytoplasmic P21 expression (Figs. 5c, d, *P* < 0.05). Oridonin intensified cytoplasmic P21 expression, as shown by immunofluorescence staining (Figs. 5e, f, *P* < 0.05). In accordance with the in vivo manifestation, P21 expression was down regulated in NRCMs with the treatment of Ang II (Figs. 5g, h, *P* < 0.05). The P21 immunofluorescence of cells also showed cytoplasmic aggregation with the treatment of oridonin, especially after AngII stimulation (Figs. 5i, j, *P* < 0.05).

### Oridonin protected myocyte hypertrophy in a P21-dependent autophagy manner in vitro

**Fig. 6:**
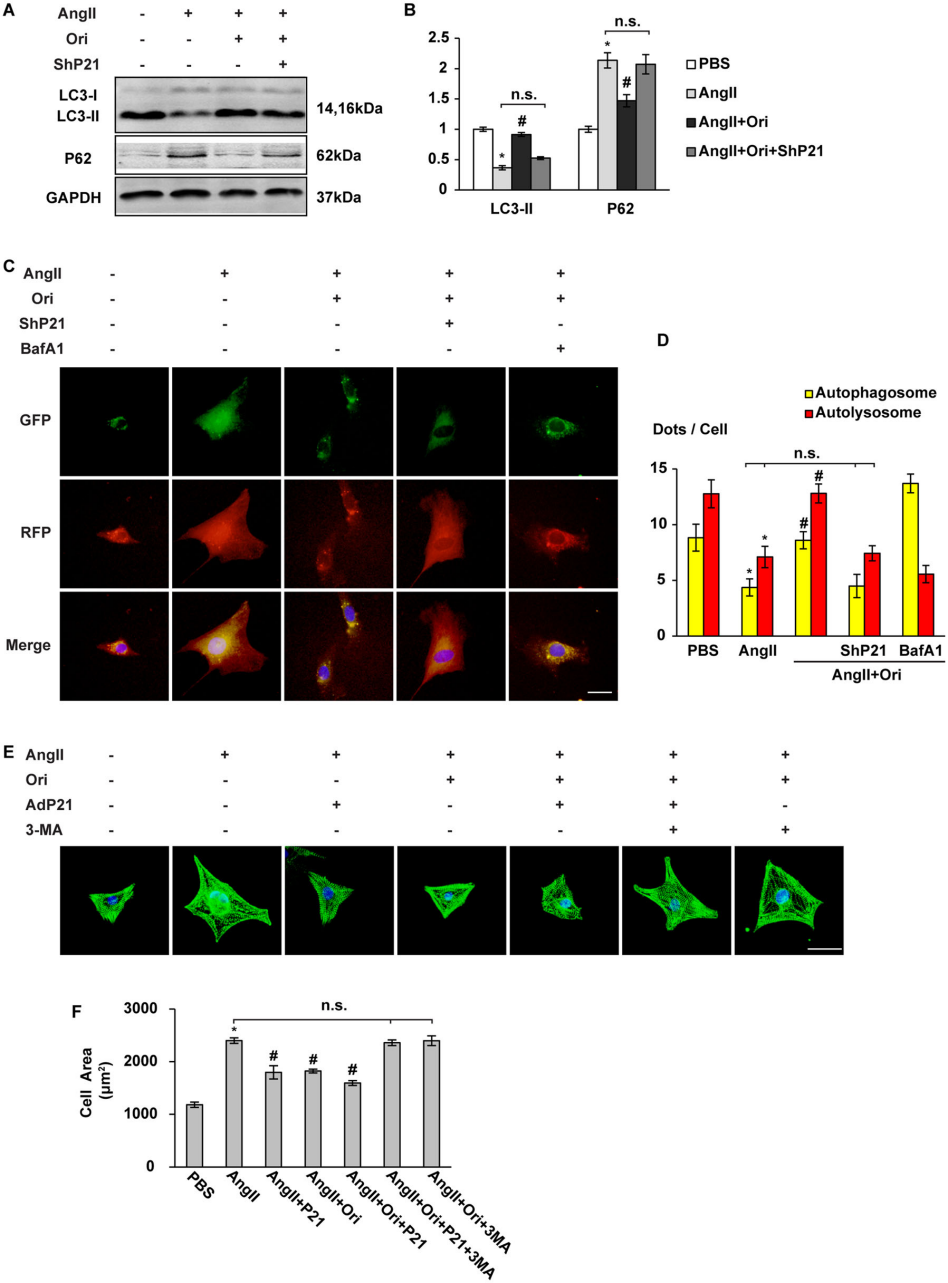
The effects of oridonin were blocked by P21-deficiency or autophagy decay in vivo. **a**, **b** Representative Western blot analyses of LC3-I/II and P62 (*n* = 6). **c**, **d** Representative fluorescence images of cardiomyocytes transfected with Cherry-red fluorescent protein (RFP)-green fluorescent protein (GFP)-LC3 adenovirus 2 h after Ang II treatment. Autophagosome (yellow dots) and autolysosome (red dots) numbers in cardiomyocytes after Ang II (2 h)/PBS treatment with or without BafA1 (100 nM) were calculated. (scale bar: 20 μm, *n* = 30 cells per group). **e** Representative images of a-actinin staining of the cardiomyocytes. P21 adenovirus-infected cells were stimulated with Ang II for 2 h, and then treated with oridonin with or without 3-MA (5 mM) as indicated. (scale bar: 20 μm). **f** Statistical results of cell area by measuring random cells (*n* = 30 cells). *P < 0.05 versus PBS. #*P* < 0.05 versus Ang II. *n.s.* not significant

To investigate whether the increase of P21 is a phenomenon accompanied by elevated autophagy, or the critical factor account for autophagy enhancement, we evaluated the specific role of P21 in cardiomyocytes by infecting NRCMs with lentivirus harbouring P21 short hairpin RNA (lentivirus-shP21, ShP21) or recombinant adenoviruses expressing murine P21 (AdP21) (Fig. S3). Myocyte hypertrophy was induced by Ang II administration. Autophagy was measured by immunoblotting of related proteins and cellular autophagic flux 2 h after Ang II treatment. Autophagic protein degradation was notably increased with oridonin treatment, but stayed suppressed with ShP21 transfection, as shown by decreased LC3II and increased P62 accumulation (Figs. 6a, b, *P* < 0.05). An mCherry-GFP-LC3 autophagic flux reporter assay was carried out to identify the specific point within the autophagic cascade inhibited by oridonin and P21. Autophagosome formation and autolysosome acidification, as shown by the yellow dots and red dots, respectively, both decreased after Ang II stimulation, which was reversed by oridonin. However, the comparably increased number of autophagosomes and autolysosomes brought by oridonin treatment were mostly reduced upon silencing of P21. We further used bafilomycin A1 (BafA1), which inhibits autophagosome-lysosome fusion, to block autophagic flux by inhibiting autolysosome turnover. Together with the oridonin promoted P21, BafA1 remarkably raised the number of autophagosomes (yellow) while depressed the formation of autolysosomes (red) (Figs. 6c, d, *P* < 0.05). These data suggested that oridonin increased myocyte autophagy by promoting autophagosome formation, and P21 was required in this process.

We next tested autophagy in cells overexpressing P21 and treated with Ang-II. H9C2 cells were infected with AdP21. LC3II immunofluorescence indicated that autophagy was suppressed in Ang II stimulated cells. In Ang II cells protected with oridonin, LC3II expression was significantly increased, showing that P21 activation is sufficient to increase autophagy in Ang II-treated cells (Fig. S4, *P* < 0.05).

We further tested whether autophagy inhibition attenuates the beneficial effects of oridonin-P21. The cellular surface areas of cardiomyocytes were measured by immunostaining with α-actinin 24 h after Ang II treatment. Autophagy inhibition by 3-MA eliminated the protective effects of oridonin on myocyte enlargement, even in the presence of additional expression of P21 by AdP21 transfection (Figs. 6e, f, *P* < 0.05).

From these data, we can infer that oridonin can inhibit the hypertrophic growth of isolated myocytes induced by Ang II in vitro. The protective effect was exerted in a P21-dependent autophagy manner.

### Manipulating P21 levels affects the anti-oxidation role of oridonin

**Fig. 7:**
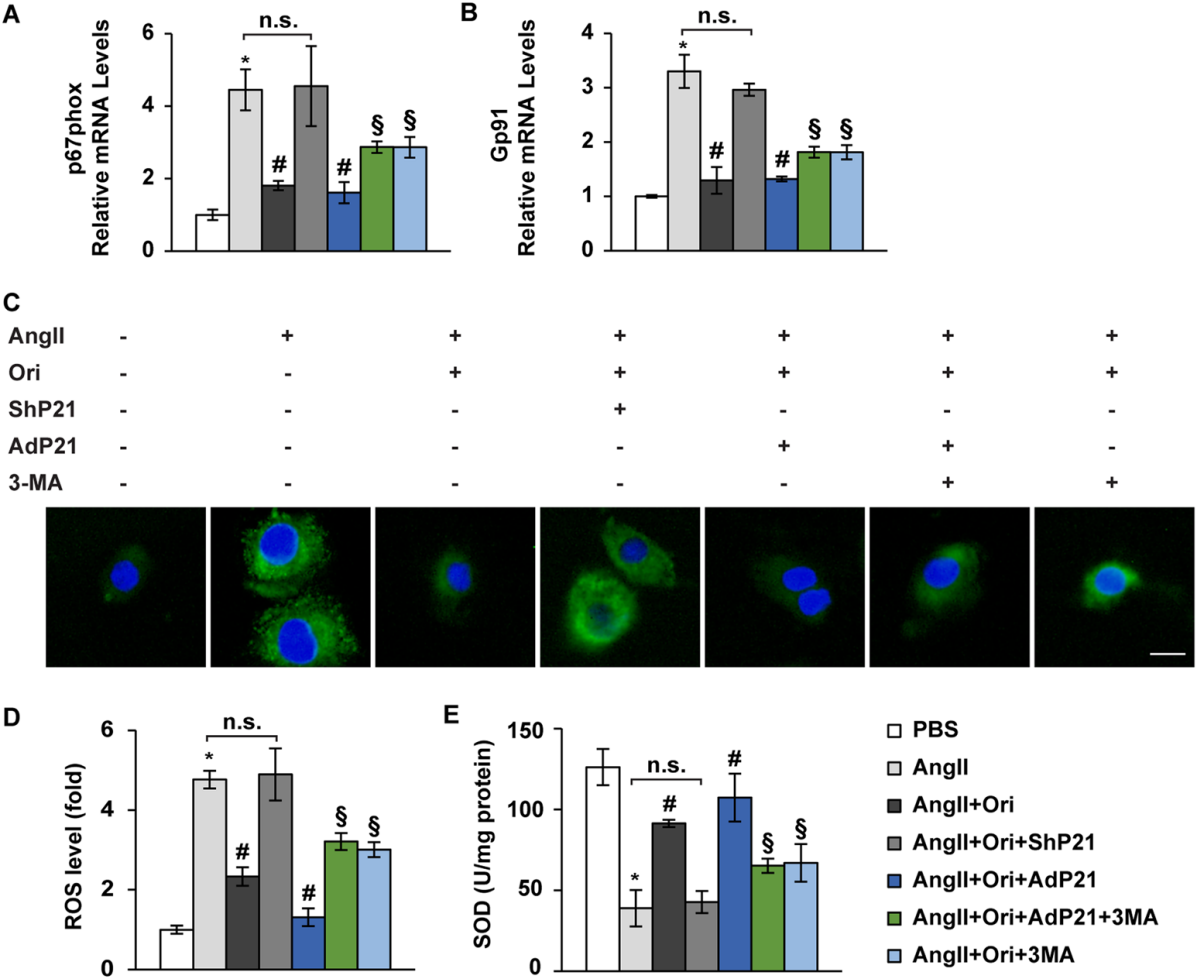
Oridonin mediated protective effects on oxidative injury beyond autophagy via P21. **a**, **b** Real-time PCR analyses of p67phox and Gp91phox (*n* = 6). **c**, **d** DCF fluorescence images and the statistical results (*n* = 6), which showed intracellular ROS in cardiomyocytes transfected with indicated virus and stimulated with Ang II for 12 h with or without oridonin. 3-MA was added 2 h before incubation with DCFH-DA probe. **e** Total activity of the antioxidant SOD in cell lysate (*n* = 6). *P < 0.05 versus PBS. #*P* < 0.05 versus Ang II. n.s. not significant. §*P* < 0.05 compared with Ang II or Ang II + Ori

Previous studies have suggested that promoting autophagy could alleviate oxidation in the heart^23^. Thus, we hypothesized that the oridonin-P21-autophagy cascade could prevent the heart from peroxidation injury. To test these hypotheses, we evaluated the effect of oridonin on oxidase expression and ROS generation in cardiomyocytes. With Ang II and oridonin treatment, NRCMs were infected with ShP21 or AdP21 to test the requirement of P21, while 3-MA was added to verify the role of autophagy. As expected, the p67phox and Gp91 levels were unresponsive to oridonin upon silencing of P21, while unexpectedly, obstruction of autophagy by 3-MA only partly disturbed the protective effects of oridonin on oxidase levels in the presence of P21 (Figs. 7a, b, *P* < 0.05). Consistently, more ROS production was detected with deficient P21 expression, while restrained autophagy partly removed the protection of oridonin on myocyte oxidative stress (Figs. 7c, d, *P* < 0.05). A converse variation tendency was found in SOD levels in cell lysate (Fig. 7e, *P* < 0.05).

### P21-deficient mice are unreceptive to oridonin action

**Fig. 8:**
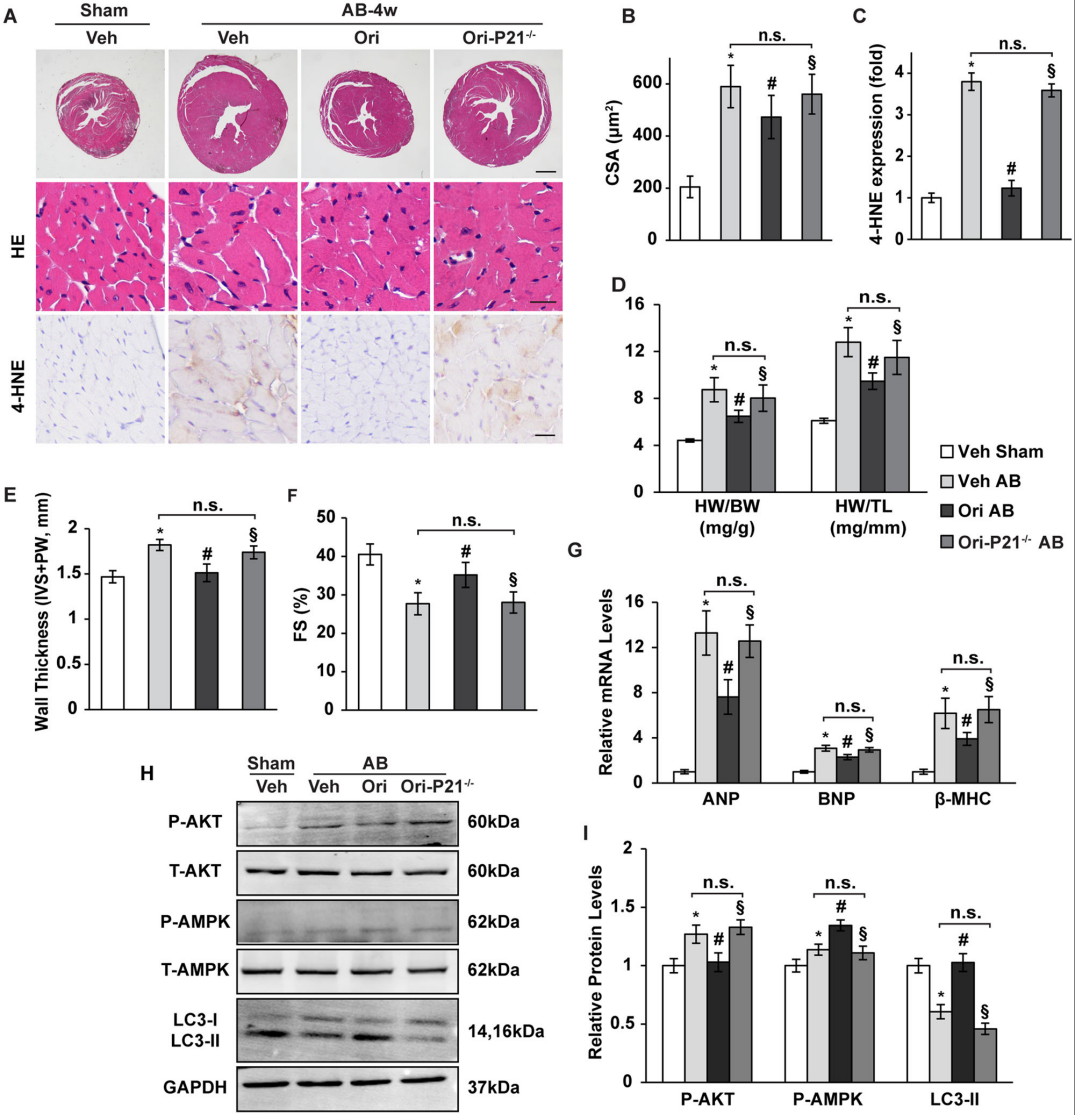
Manipulation of P21 levels in mice affects the antihypertrophic effect of oridonin. **a** Histological analysis of whole hearts and heart sections stained with H&E (the upper and middle panel, scale bar: 1000 μm and 20 μm, *n* = 6). Representative immunohistochemical analysis of 4-HNE (the lower panel, scale bar: 20 μm, *n* = 6) in mouse hearts from the indicated groups 4 weeks after AB or sham surgery. **b** Statistical results for the cross-sectional areas and 4-HNE fold change in mouse hearts (*n* = 100 cells). **c** Statistical results of HW/BW and HW/TL (*n* = 6). **d**, **e** Summarized echocardiographic measurements of diastolic wall thickness [interventricular septum (IVS) + posterior wall (PW)] and fraction shortening (FS). **f** Real-time PCR analyses of the transcriptional levels of ANP, BNP, and β-MHC (*n* = 6). **g**, **h** Representative Western blot analyses of P-AKT, T-AKT, P-AMPK, T-AMPK and LC3-I/II (*n* = 6). Data are represented as the means ± SD. *P < 0.05 versus vehicle-sham. #*P* < 0.05 versus vehicle-AB. §*P* < 0.05 versus AB-oridonin. *n.s.* not significant

To further confirm whether oridonin exerted antihypertrophic effects through P21 in vivo, we generated viable global P21 knockout mice (hereafter called P21^−/−^ mice). P21^−/−^ mice and their wild-type littermates were subjected to AB surgery, and 40 mg/kg of oridonin was given orally once a day for 4 weeks. P21 knockout abolished oridonin-induced cardiac hypertrophy and oxidative injury (Figs. 8a–c, *P* < 0.05). The P21 dependent protective properties in the heart were also evidenced by increased heart weight and ventricular wall thickness, as well as decreased fractional shortening (Figs. 8d–f, *P* < 0.05). The role of increased autophagy mediated by oridonin-P21 was investigated by autophagy inhibitor in vivo. Intraperitoneal injection of 3-MA resulted in an increased HW/BW and left ventricular wall thichness (IVS + PW), and reduced FS in mice (Fig. S5, *P* < 0.05). Transcript levels of hypertrophic markers ANP, BNP and βMHC were also elevated in P21^−/−^ mouse hearts (Fig. 8g, *P* < 0.05). Additionally, P21 deficiency altered AKT/AMPK phosphorylation and LC3 membrane transfer via oridonin therapy (Figs. 8h, i, *P* < 0.05).

## Discussion

In the present study, we investigated the effects of oridonin on left ventricular remodelling after AB-induced chronic pressure overload. We provided evidence of oridonin as a potent anti-oxidant with P21/autophagy-augmenting properties.

**Fig. 9:**
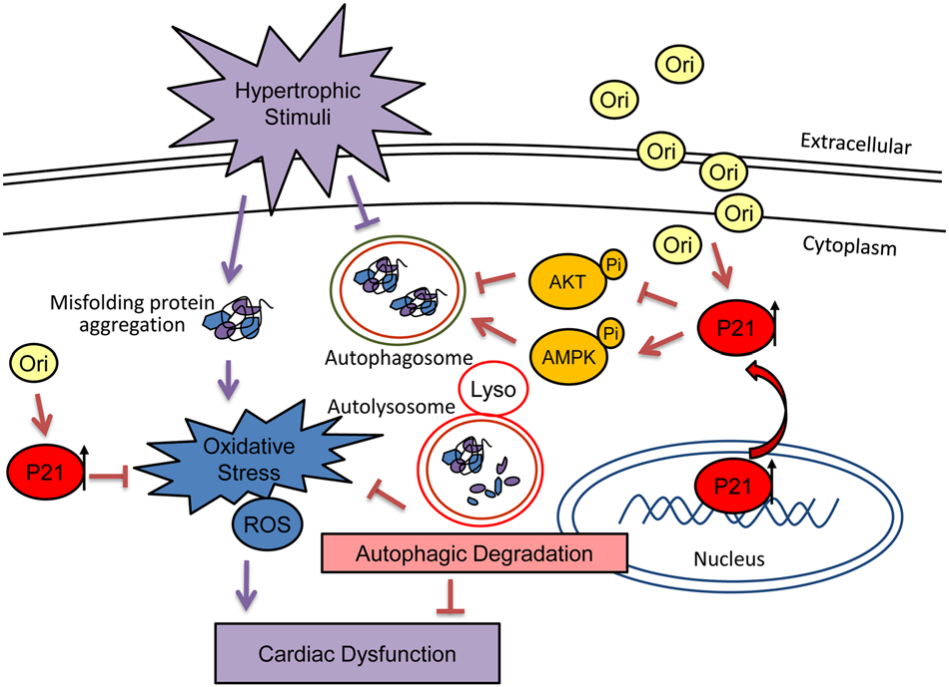
Working model. Oridonin, by promoting cytoplasmic P21, activates cardiomyocyte autophagic flux while reducing reactive oxygen species production and preventing cardiac injury during cardiac hypertrophy

Our study involving AB-induced ventricular hypertrophy in vivo and angiotensin-induced hypertrophic responses of cardiomyocytes in vitro showed that oridonin treatment significantly protected the heart from pathological remodelling and dysfunction. These beneficial effects were related to alleviating cardiac hypertrophy, fibrosis, and oxidative stress. Consistent with our hypothesis, oridonin remarkably activated P21-induced autophagy in the heart, and its cardioprotective properties were blunted with either a genetic disturbance of P21 or inhibition of autophagy, suggesting that the P21-promoted autophagy mediates the salutary effects of oridonin. Moreover, interference with the P21 level affects the elimination of oxygen free radicals by oridonin independent of the autophagy process, which implies that P21 possessed antioxidation properties under oridonin treatment (Fig. 9). This is the first report to demonstrate that oridonin can obstruct cardiac hypertrophy and activate autophagy via P21. Our findings suggestively extend previous evidence establishing that oridonin protects cells in response to stress, indicating that oridonin could be a promising therapeutic agent against cardiac hypertrophy.

Our findings in AB-induced cardiac hypertrophy expand previous evidence that oridonin protects against stress injury, which offers new approaches into the administration of oridonin to defend myocardial dysfunction. Oridonin has long been characterized as a complex ent-kaurane diterpenoid that exhibits remarkable antitumour and antitoxic effects^24,25^. Studies have documented the antioxidant and anti-fibrosis activities, as well as the cardiac distribution of oridonin^26–28^, which suggested a potential protective role of oridonin under cardiovascular stress. However, oridonin has not hitherto been applied in pathological cardiac hypertrophy or other cardiovascular diseases. In our study, as expected, oridonin exerted a protective effect against the development of cardiac hypertrophy as revealed by mitigated myocytes enlargement, alleviated fibrosis, and restricted oxidative injury. However, the detailed mechanism or cellular target that underlies the antihypertrophic activity of oridonin remains obscure.

Previous studies implied that the beneficial effects of oridonin might be mediated by autophagy activation, which offered an important source of ATP and could inhibit the generation of reactive oxygen species^19,20,29^. In the setting of the heart under stress, emerging evidence has demonstrated that impaired myocardial autophagy, being unable to breakdown intracellular aggregates, played crucial roles in the development of cardiac hypertrophy and HF^21,30^. Pharmacological interventions targeting the autophagosome-lysosome pathway, meanwhile, ameliorated cardiac remodelling^11,14,31^. In this study, we presented both in vivo and in vitro evidence that the protective effects of oridonin on cardiac hypertrophy were mediated through motivation of autophagy, as oridonin (1) facilitated the formation of LC3-positive autophagosomes and (2) coordinated the core molecular machinery ATG proteins covering the fusion and maturation and degradation of autophagosomes; (3) its protective effects on myocyte hypertrophy were eliminated by autophagy inhibition using 3-MA. These results were consistent with previous studies implying the autophagy-inductive action of oridonin^19,20,29^. Moreover, we found that oridonin blunted the phosphorylation of AKT and mTOR while salvaging the phosphorylation of AMPK. Autophagy is regulated by AKT-mTOR and AMPK-ULK1 signalling, which activates the anabolic and catabolic processes respectively, and interact to control autophagosome formation^32^. Sustained pressure overload induces long-term activation of Akt, which successively activates mTORC1 to accentuate cardiac contractile defects through a loss of autophagic regulation^33^. AMPK serves as a sensor and regulator of cellular energy status and can promote autophagy^34^. Our data showing that oridonin treatment attenuated maladaptive hypertrophy via AMPK were consistent with a previous report that AMPK prevents heart failure through enhancing autophagy^35^. Thus, oridonin exerts its antihypertrophic effect throughout the classic autophagy pathway. However, contrary to our present finding, AMPK was deactivated following oridonin treatment in cancer cells^20^. Our data implied that oridonin had diverse regulatory effects on AMPK in different cell types. Nevertheless, the molecular target in cardiomyocytes by which oridonin induces autophagy has not yet been identified.

Previous research suggested that the beneficial effects of oridonin might be mediated by the activation of P21^36^. Our study probes into the role of P21 in oridonin-induced autophagy. Here, the conceptually important points that emerge from our study are (1) oridonin intensified cytoplasmic P21 expression; (2) oridonin failed to inhibit myocyte hypertrophy in either P21 silenced NRCMs or P21^−/−^ mice (Figs. 6 and 8); and (3) oridonin action on myocyte enlargement is lost when autophagy is blocked by 3-MA in the presence of P21. These findings indicate that the upregulation of P21 might be an initial response to oridonin treatment in cardiomyocytes. Cardiac myocytes are regarded as terminally differentiated cells. Thus, the cyclin-dependent kinase inhibitor P21 exerts important functions in the regulation of growth (hypertrophy) related processes^37^. A critical role for P21 in the statin-dependent inhibition of cardiac hypertrophy has implied the potential therapeutic importance of P21 for the prevention of heart failure^38^. Upregulated expression of P21 following oridonin treatment could induce autophagy in human prostate cancer cells, which provide potential mechanisms of P21 being a therapeutic target^22^. The essential autophagy gene Atg7 could regulate the transcription of the gene encoding P21^39^. Hence, the relationship between autophagy and P21 remains to be clarified. According to our observation, P21 serves as a critical factor accounting for the oridonin-induced autophagy enhancement. Moreover, we used BafA1, an inhibitor of late-stage autophagy, to distinguish between activation of autophagosome formation or the autophagic vacuole processing action of P21. The results suggested that increases in steady-state LC3-II levels may derive from directly controlling the activation of autophagic flux by oridonin and the upregulated P21. The oridonin-P21-autophagy cascade could inhibit misfolded proteins escaping from impaired autophagy and triggering cardiac peroxidation injury^21^.

Although our study focused on the autophagy promoting effects of P21 with oridonin treatment, as regards cardiomyocyte hypertrophy, P21 may also have other regulatory properties. Notably, 3-MA blocked the protection of oridonin on myocyte enlargement but allowed its alleviation of peroxidation injury. In line with these observations, the role of P21 as a suppressor of cardiac hypertrophy or oxidative stress was highlighted in several studies^37,40,41^. Taking all the findings into consideration, we surmise that oridonin-mediated alleviation of cardiac hypertrophy is a consequence of autophagy activation and oxidation inhibition caused by increased P21 expression.

The strengths of our study include the use of oridonin on cardiac hypertrophy in vivo and in vitro, the evaluation of the protective effects of oridonin on the accompanying pathological manifestations, the determination of a possible mechanism of oridonin action, and the identification of a potentially useful molecule that mediates the pharmacological function. The findings from our study provided evidence for the application of oridonin in the treatment of pressure overload-induced cardiac hypertrophy, which may have pragmatic implications in the future. Finally, although we clearly demonstrated that oridonin-P21 induced autophagy are responsible for the protection of cardiac hypertrophy, the gap of how oridonin modificate P21, and lead to activation of autophagy needs to be further filled.

## Clinical perspectives

Impaired autophagy and oxidative injury are closely involved in cardiac remodelling. We investigated whether the traditional Chinese medicine oridonin could inhibit these hypertrophic responses.

We found that oridonin protected the heart from pressure overload induced hypertrophy by promoting the P21-related autophagy pathway.

Our study provided evidence for the application of oridonin in the therapy of cardiac hypertrophy.

## Materials and methods

### Chemicals

Oridonin (Ori, 98% purity) was purchased from Shanghai Winherb Medical Science Co., Ltd. (Shanghai, China).

### Cell cultures and treatments

To examine the in vitro anti-hypertrophic effects of oridonin, neonatal rat cardiac myocytes (NRCMs) were isolated and cultured in Dulbecco’s modified Eagle’s/F-12 (11330, Gibco, Grand Island, NY, USA), supplemented with 15% foetal bovine serum (10099, Gibco) as previously described^42^. H9C2 cells were obtained from the Cell Bank of the Chinese Academy of Sciences (Shanghai, China) and were cultured in DMEM (11885, Gibco) with 10% FBS. Cells were incubated in a humidified incubator (SANYO 18M, Osaka, Japan) at 37 °C with 5% CO_2_. To simulate in vitro myocardial hypertrophy, the cells were stimulated with Angiotensin II (Ang II, 1 μM, A9525, Sigma-Aldrich, St. Louis, MO, USA), in the presence or absence of different concentrations of oridonin (dissolved in 0.1% DMSO PBS at a concentration of 5, 20, and 50 μM), and incubated for another 12 or 24 h.

### Cardiomyocytes Immunofluorescence analysis

The cell surface area of the NRCMs was assessed via immunofluorescence staining. Briefly, the cardiomyocytes were fixed with 3.7% formaldehyde, and permeabilized with 0.1% Triton X-100 in PBS, followed by staining with α-actinin (3134, Cell Signaling Technology, CST, Danvers, MA, USA) overnight at 4 °C. The cells were subsequently incubated with Alexa Fluor 488 (green) secondary antibodies (1:200) for 60 min at 37 °C, followed by the visualization of nuclei with 4,6-diamidino-2-phenylindole (DAPI). The surface areas were measured using Image-Pro Plus software, version 6.0. At the magnification of 400 times, 6–10 microscopic vision were randomly selected and 5 cells were counted for each vision (30–50 cells were counted for each group). AOI was used to automatically trace the cell boundary, and the cell area was converted by the tool COUNT/SIZE according to the ruler taken under the same magnification.

### Ethical statement

All of the animal care and experimental procedures were performed in accordance with the Guidelines for the Care and Use of Laboratory Animals, published by the US National Institutes of Health (NIH Publication, revised 2001), and were approved by the Animal Care and Use Committee of Renmin Hospital of Wuhan University.

### Animals

Male, 8–10 weeks old mice in the C57BL/6 background (body weight ranging from 23.5 to 27.5 g) were purchased from the Institute of Laboratory Animal Science, Chinese Academy of Medical Sciences (Beijing, China). Mice with targeted knockout mutation of the gene encoding P21^CIP1/WAF1^ (016565, B6.129S6-Cdkn1a^tm1Led^/J) were purchased from The Jackson Laboratory (Bar Harbor, USA).

### Aortic banding animal models

To establish a mouse model of cardiac hypertrophy, aortic banding (AB) surgery was performed to create pressure overload-induced hypertrophy. Animals were weighed, randomly divided into groups, and then received the surgical procedure, during which mice were anaesthetized (3% sodium pentobarbital, 40 mg/kg) and AB or sham surgery was performed as previously described^43^. The next day, mice were administered oridonin suspension (40 mg/kg) or equal volumes of vehicle by gavage. One, 2 and 4 weeks post AB surgery, the mice were sacrificed by cervical dislocation, and the left ventricular tissue was excised for further detection. The hearts and lungs were weighed to calculate the heart weight/body weight ratio (HW/BW, mg/g), lung weight/body weight ratio (LW/BW, mg/g) and HW/tibia length (HW/TL, mg/mm) in each group 4 weeks post AB surgery.

### Echocardiography and haemodynamics

To inspect the cardiac function, echocardiography and haemodynamic monitoring were analysed 4 weeks after AB surgery, as described previously^44^. Mice were anaesthetized by 1.5% isoflurane, while the inhalational flow was adjusted to maintain their heart rate at 450–550 beats/min. Transthoracic ultrasonography was performed with a MyLab 30CV ultrasound system (Biosound Esaote, Florence, Italy). The left ventricle (LV) was assessed by M-mode scanning in both parasternal long- and short-axis views at the mid-papillary muscle level. LV internal dimensions and chamber wall thickness at diastole and systole were measured respectively. Invasive haemodynamic monitoring was implemented with a microtip catheter transducer (SPR-839, Millar Instruments, Houston, TX, USA), which was inserted into the right carotid artery and proceeded into the LV. Pressure signals and volume signals were continuously recorded with a Millar Pressure-Volume System (MPVS-400, Millar Instruments) coupled with a Powerlab/4SP A/D converter and then stored and displayed. The data were processed using PVAN data analysis software.

### Cardiac morphology and histomorphometric analysis

To assess cardiac hypertrophy and fibrosis, we performed morphological analysis on heart sections. Mouse hearts were excised after euthanasia and immediately placed into 10% potassium chloride solution to be arrested in diastole. Subsequently, these hearts were fixed in 10% formalin for 12–24 h, embedded in paraffin, and then sectioned transversely at 3–5 µm. Afterward, the sections were stained with haematoxylin-eosin (HE) or picrosirius red (PSR) to evaluate myocyte cellular hypertrophy or extracellular collagen deposition, respectively. The cross-sectional areas of myocytes and fibrotic areas were measured using a digital image analysis system (Image-Pro Plus, version 6.0) from captured images of the stained sections.

### Immunofluorescence and immunohistochemistry

Immunofluorescence staining was performed in order to determine the expression as well as the localization of LC3/P21 in the mice hearts. Briefly, for the heart section staining, all sections were autoclaved with citrate solution for antigen retrieval. After blocking with 10% goat serum (GTX27481; GeneTex, Sanantonio, TX, USA), the sections were incubated in LC3A/B (12741, CST) or P21 antibody (sc-6246, Santa Cruz Biotechnology, Dallas, TX, USA) overnight. Heart sections were subsequently incubated with secondary antibodies for 60 min at 37 °C, followed by visualization of the nuclei with DAPI. For immunohistochemistry, the tissue sections were heated for antigen retrieval, and then incubated with anti-heme oxygenase-1 (ab13243, Abcam, Cambridge, UK) or 4-hydroxynonenal (ab46545, Abcam), followed by incubation with goat anti-rabbit EnVisionTM+/HRP reagent, and finally staining with a DAB detection kit. The images were captured using a fluorescence microscope (OLYMPUS DX51, Tokyo, Japan) and then analysed using Image-Pro Plus software.

### Quantitative real-time RT-PCR

To examine the mRNA expression of cardiac hypertrophy and fibrosis related markers, total mRNA was extracted from the mouse LV using TRIzol reagent (15596-026, Invitrogen, Carlsbad, CA, USA) according to the manufacturer’s instructions and subsequently reverse transcribed into cDNA using oligo (DT) primers and the cDNA Synthesis Kit (4897030001, Roche, Basel, Switzerland). The indicated genes were amplified using LightCycler 480 SYBR Green 1 Master Mix (04887352001, Roche), and the expression levels of target genes were normalized to GAPDH gene expression.

### Western blot

Western blot analysis was performed to evaluate protein expression. Total proteins from mouse hearts or cultured cardiomyocytes were extracted by use of RIPA lysis buffer supplemented by a protease inhibitor cocktail (Complets, Roche). Cytoplasmic proteins were extracted using an extraction kit (C500051, Sangon, China). Protein concentration was evaluated using a BCA protein assay kit (23227; Thermo Scientific, Waltham, MA, USA). Equal amounts (50 μg) of protein were electrophoresed through 10% SDS-PAGE and transferred to PVDF membranes (IPFL00010, Millipore, Billerica, MA, USA). Next, the membranes were blocked with 5% skimmed milk at room temperature and then incubated overnight at 4 °C with indicated primary antibodies against different antigens, including P21 (ab109199, Abcam), SOD2 (ab38155, Abcam), HO-1 (ab13243, Abcam), P-AKT (4060, CST), T-AKT (4691, CST), P-mTOR (2971, CST), T-mTOR (2983, CST), P-AMPK (2535, CST), T-AMPK (2603, CST), LC3 (12741, CST) and P62 (23214, CST). Protein expression levels were normalized to that of GAPDH (2118, CST). Secondary antibodies were incubated at room temperature for 1 h the next morning. Finally, images were obtained by Odyssey Infrared Imaging System (LI-COR Biosciences, Lincoln, NE, USA) to quantify protein expression.

### Recombinant viral vectors and infection

To knock down P21 expression, NRCMs were infected with lentiviral particles containing the short hairpin RNA targeting P21 (Lenti-shP21, sc-108036-V, Santa Cruz Biotechnology). To overexpress P21, an adenovirus vector carrying the P21-coding gene (Ad-P21) were generated by iBioscience Company (Jinan, China). NCRMs were infected with the virus at a multiplicity of infection of 100 for 8 h. Lenti-shNC and AdNC were used as non-targeting controls.

### Oxidative stress detection

To assess total superoxidase dismutase (SOD) and glutathione peroxidase (GPx) activity and the content of malondialdehyde (MDA) and dihydroethidium (DHE) in the myocardium, a series of commercial assay kits (Beyotime, Shanghai, China) was obtained and performed according to the manufacturer’s instructions. Briefly, heart tissues were lysed, centrifuged, and then mixed with the detection working dilution. A microplate reader (BioTek Instruments, Inc., US) was used to record the luminescence, and then SOD, GPx and MDA content was calculated. ROS generation was detected by DHE staining in vivo and DCFH-DA staining in vitro respectively. In brief, NRCMs were incubated with DCFH-DA (5 μM) at 37 °C for 30 min in a dark chamber. Fluorescent images were observed with a fluorescence microscope (Olympus DX51).

### Transmission electron microscopy

Heart sections were viewed by transmission electron microscopy to detect autophagosomes and autolysosomes. Freshly isolated hearts were fixed with 2.5% glutaraldehyde in PBS (pH 7.0) at 4 °C for 8 h and then post-fixed for 1 h in 1% OsO_4_. After being dehydrated and embedded, tissues were cut into ultrathin sections (70–100 nm) using an ultramicrotome (Leica EMUC6, Wetzlar, Germany) and stained with uranyl and lead citrate. Finally, image observation was performed using a transmission electron microscope (H-7650B; Hitachi Limited, Tokyo, Japan). Micrographs were captured at different magnifications (2000 and 8000-fold).

### Autophagic flux analysis

To assess autophagic flux in mice heart by Western blot, bafilomycin A1 (BafA1; 1.5 mg/kg IP) was administered to mice 2 h before they were euthanized. To assess autophagic flux in NRCMs, mCherry-GFP-LC3 adenovirus (Vigene, MOI = 50) was administered to infect NRCMs, and 3-methyladenine (3-MA, 5 mM, M9281, Sigma) was used to interfere autophagy initiation while bafilomycin A1 (BafA1, 2 nM, B1793; Millipore) was added to block autophagosome-lysosome fusion. For red fluorescent protein (Cherry)/green fluorescent protein (GFP) analysis, NRVMs were cultured on coverslips; after treatment with Ang II, 3-MA or BafA1 for 2 h, cells were washed with ice-cold PBS, and fixed with 4% paraformaldehyde, and then scanned with a fluorescence microscope.

### Statistical analysis

All statistical analyses were computed using the SPSS 22.0 software and the results are presented as the means ± SD. All measurement data had normal distributions (*P* > 0.05) according to the one‑sample K‑S test. One-way analysis of variance (ANOVA) was used to evaluate differences between multiple groups, followed by a post hoc Tukey test. Comparisons between two groups were assessed by Student’s *t*-test. *P* < 0.05 was defined as statistically significant.

## Supplementary information

Supplementary material
