# Supplementary material for: Oridonin protects against cardiac hypertrophy by promoting P21-related autophagy
Source: Cell Death Dis. 2019 May 24;10(6):403. doi: 10.1038/s41419-019-1617-y (PMC6534559; doi:10.1038/s41419-019-1617-y)
Supplement: Supplementary file 1 — Supplementary material [file 41419_2019_1617_MOESM1_ESM.doc]

**
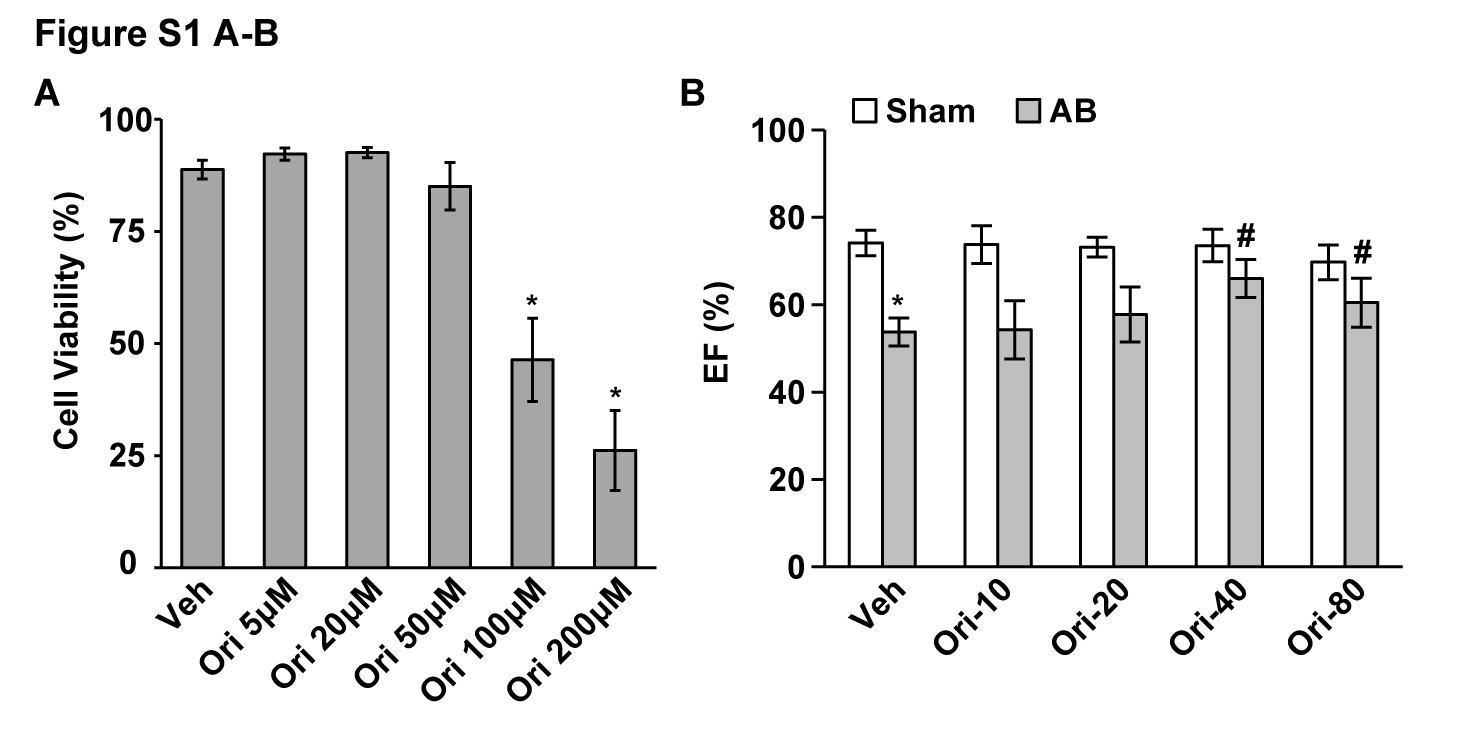
**

**Figure S1.** A. H9C2 cells were treated with different concentrations of oridonin (5, 20, 50, 100, and 200 µM) for 24 hours. Cell viability was measured by ECCK‑8 assay. *P<0.05 vs. vehicle (0 µM oridonin). B. Ejection Fraction calculated by echocardiographic parameters showed cardiac function of oridonin- treated mice 4 weeks after AB surgery (n=6). The concentration of oridonin was at 10, 20, 40, and 80 mg/kg/day, Data are represented as mean ± SD. *P<0.05 versus vehicle-sham. #P<0.05 versus vehicle-AB.

**Cell Viability Assay**

To determine the cytotoxicity of oridonin, H9C2 cells (Cell Bank of the Chinese Academy of Sciences, Shanghai, China) were cultured in a 96-well plate, treated with oridonin at concentrations of 1, 5, 20, 50, 100 and 200 μM. Cell viability was evaluated using the cell counting kit assay (Enhanced Cell Counting Kit-8, ECCK‑8, C0042, Beyotime, Shanghai, China), in accordance with the manufacturer’s instructions. The effect of oridonin on cell viability was measured by absorbance at 450 nm using a microplate reader (Synergy HT, Bio-tek, Vermont, USA) and expressed as the percentage compared with the vehicle group, which was set at 100%.


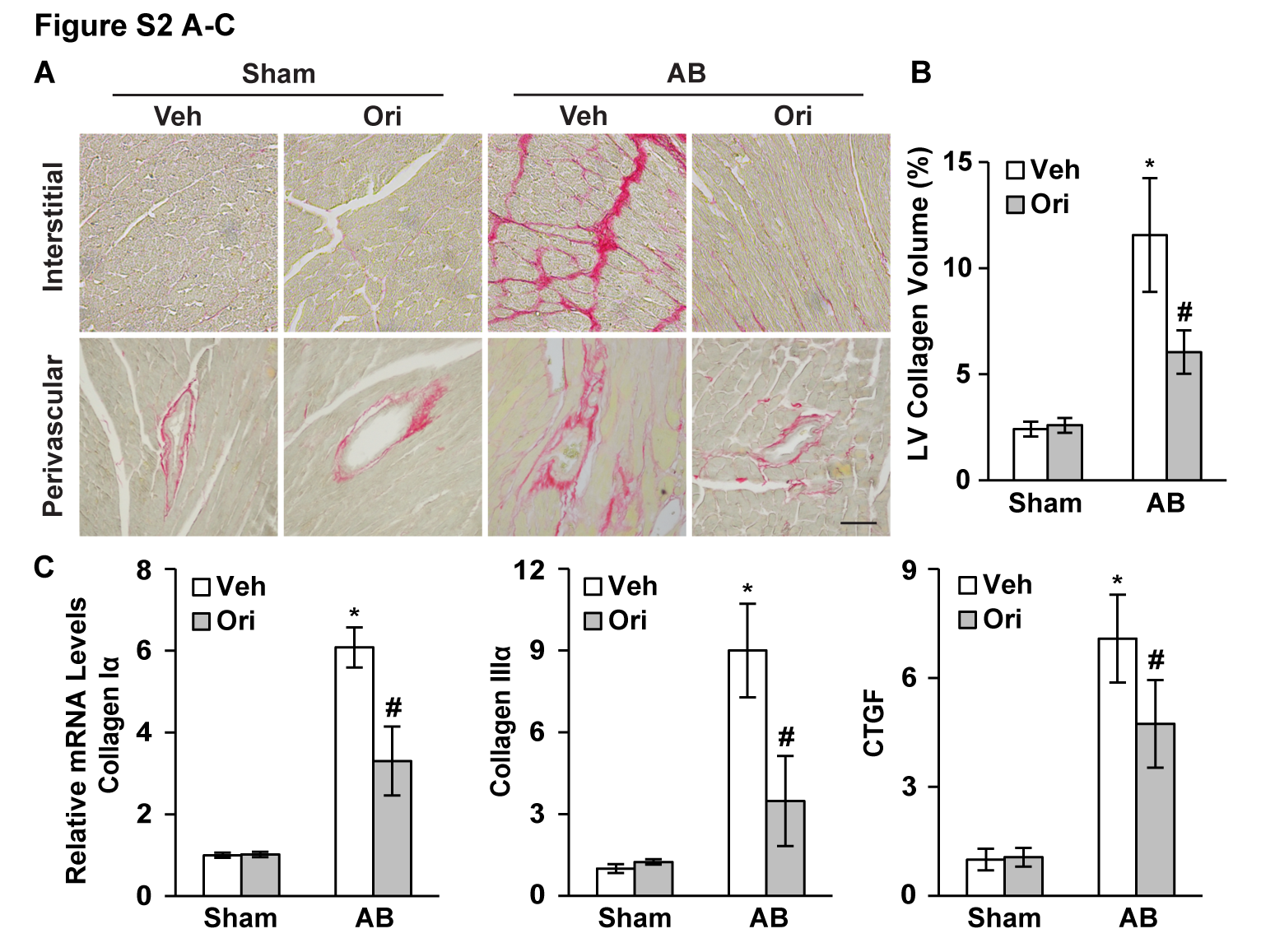


**Figure S2. Oridonin prevented pressure overload induced fibrosis.** A. Picrosirius red staining of left ventricular sections in the indicated groups (scale bar: 50 μm, n=6). B. Quantification of collagen volume (n=30 fields). C. Real-time PCR analyses of the fibrotic markers, collagen I, collagen III, and connective tissue growth factor (CTGF) (n=6). *P<0.05 versus vehicle-sham. #P<0.05 versus vehicle-AB.

**
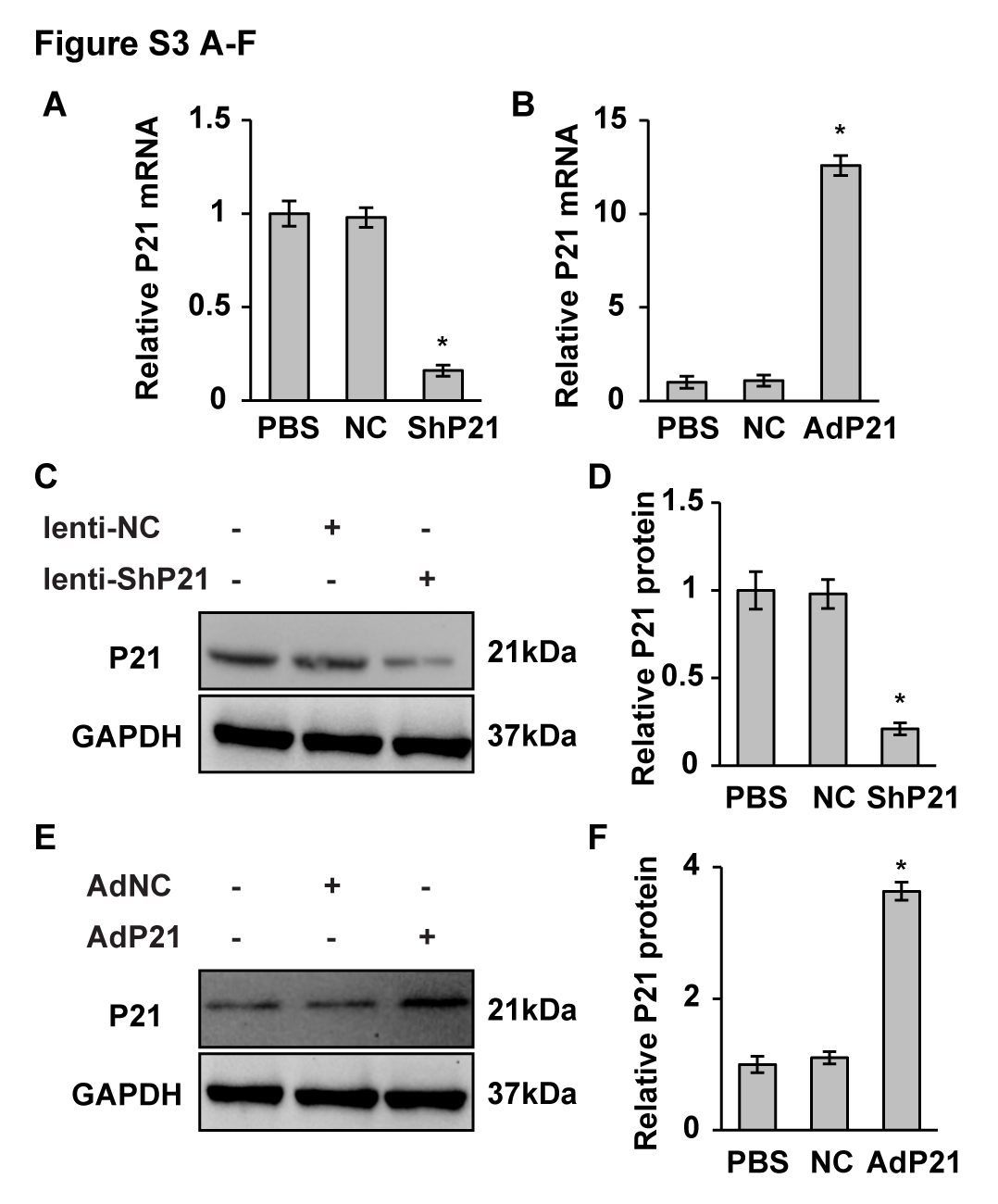
**

**Figure S3. P21 expression after virus transfection.** Primary cardiomyocytes were infected with the AdP21 virus or lenti-ShP21 virus for 24h. Control vector AdNC or lenti-NC were used to exclude the interference of the virus vectors. The infectious efficiencies of the virus were assessed by RT-PCR and western blot. A-B. The relative mRNA expression level of P21 in NRCMs (n=6). E-F. Immunobloting analysis of P21 protein levels (n=6). *P<0.05 versus PBS.


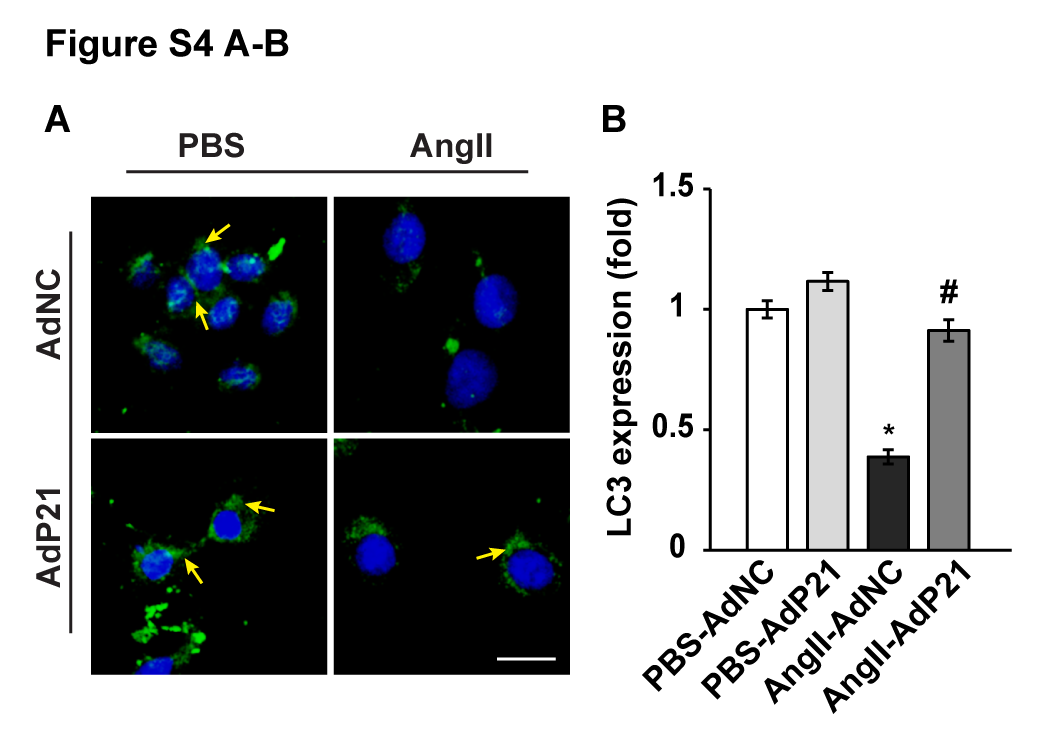


**Figure S4.** **P21 overexpression increased autophagy in Ang II-treated cells**. Detection of LC3 expression by immunofluorescence staining in the Ang II stimulated H9C2 cardiomyocytes transfected with AdP21 or the control vector (scale bar: 20 μm, n=6). *P<0.05 versus AdNC-PBS. #P<0.05 versus AdNC-AngII.


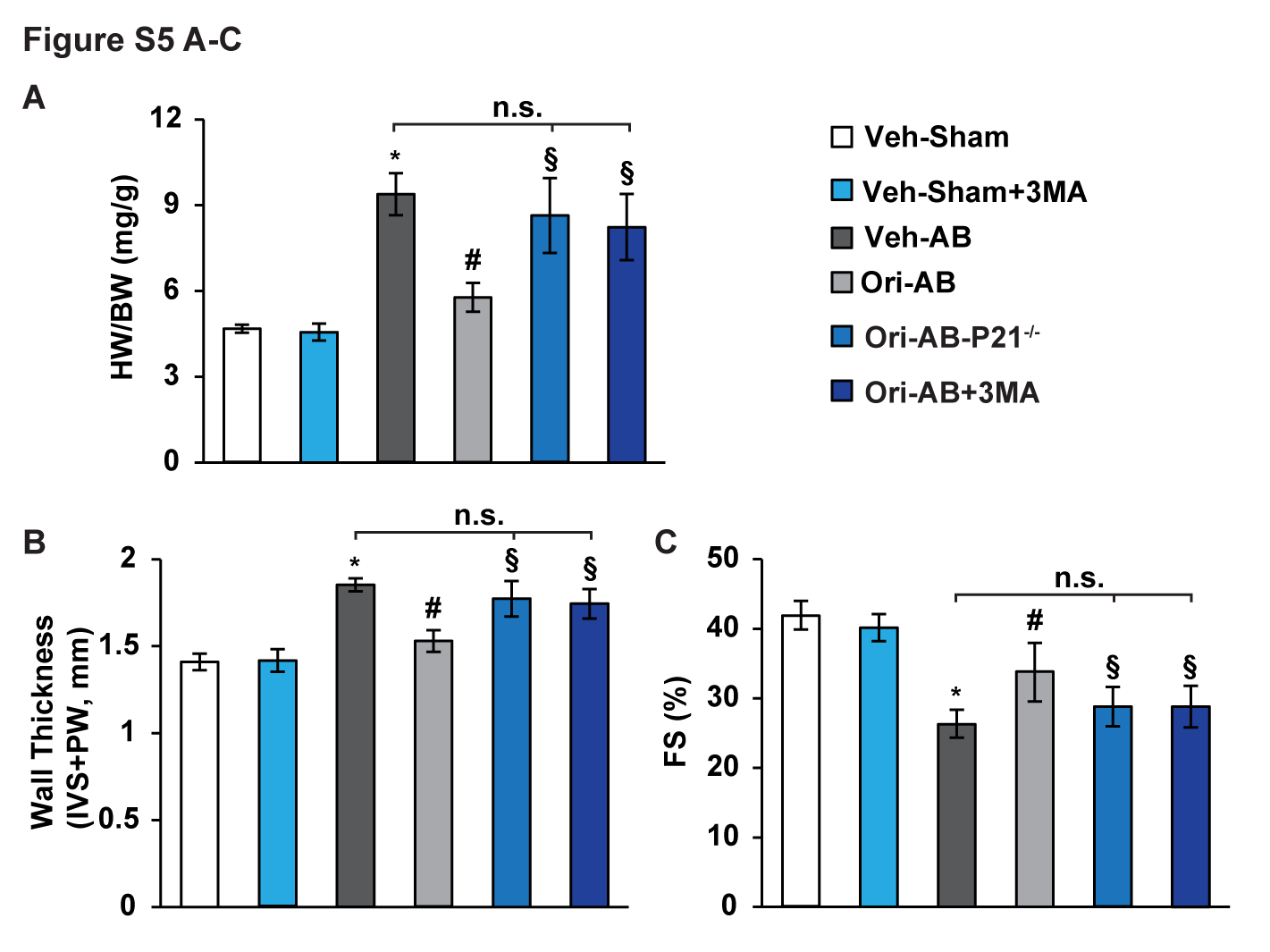


**Figure S5. Autophagy inhibition abolished the protective role of oridonin in vivo.** A. Statistical results of HW/BW and HW/TL (n=6). B-C. Statistical results of echocardiographic of diastolic wall thickness [interventricular septum (IVS) + posterior wall (PW)] and fraction shortening (FS) (n=6). *P<0.05 versus vehicle-sham. #P<0.05 versus vehicle-AB. §P<0.05 versus AB-oridonin. n.s. not significant.
